# Sex-Specific and Reproductive Status-Dependent Effects of Liraglutide on Metabolic Disorders Associated with Prediabetes

**DOI:** 10.3390/antiox15060729

**Published:** 2026-06-09

**Authors:** Lucie Lebertová, Irena Marková, Martina Hüttl, Kristýna Černá, Iveta Zapletalová, Hana Malínská

**Affiliations:** 1 Centre for Experimental Medicine, Institute for Clinical and Experimental Medicine, 140 21 Prague, Czech Republic; lucie.lebertova@ikem.cz (L.L.); irma@ikem.cz (I.M.); mabw@ikem.cz (M.H.); ceak@ikem.cz (K.Č.); 2 First Faculty of Medicine, Charles University, 128 01 Prague, Czech Republic; 3 Department of Pharmacology, Faculty of Medicine and Dentistry, Palacky University Olomouc, 779 00 Olomouc, Czech Republic; iveta.zapletalova@upol.cz

**Keywords:** GLP-1 receptor agonists, sex differences, ovariectomy, prediabetes, lipid metabolism, insulin resistance

## Abstract

Glucagon-like peptide-1 receptor agonists (GLP-1 RAs) have been shown to have beneficial effects in T2D, reducing hepatic lipid storage and improving metabolic dysfunction-associated steatotic liver disease. However, sex and reproductive age may influence their effect. We investigated the effect of liraglutide administration (0.2 mg/kg/day subcutaneously for 8 weeks) on metabolic disorders in relation to sex and reproductive age, using male, female and ovariectomized female hereditary hypertriglyceridemic (HHTg) rats as a prediabetic model. Liraglutide improved glucose tolerance in all HHTg rats. Female and ovariectomized (OVX) female rats showed a stronger effect of lipid metabolism and visceral adiposity than males. Moreover, no changes in hepatic triacylglycerol (TAG) accumulation were observed in males. Liraglutide partially reversed ovariectomy effects, such as increased body weight, visceral obesity and impaired glucose tolerance. Compared with males, female and OVX female rats showed more significant changes in hepatic gene expression involved in lipogenesis (*Scd-1*, *Srebp1*, *Pparγ*), fatty acid and lipid metabolism (*Pparα*, *Hmgcr*, *Srebp2*) and fibrosis (*Tgfβ*), which may improve hepatic lipid metabolism. Females of fertile age showed greater improvements in insulin sensitivity, reductions in ectopic lipid accumulation, and improvements in lipid metabolism. Depending on sex and reproductive status, liraglutide can mitigate fatty liver before diabetes onset.

## 1. Introduction

Metabolic dysfunction-associated steatotic liver disease (MASLD) affects more than 38% of the global population [1]. The impact is even greater among people with type 2 diabetes (T2D), nearly 70% of whom have MASLD [2]. Liraglutide belongs to the incretin class, glucagon-like peptide-1 (GLP-1) receptor agonists (GLP-1 RAs), which provide comprehensive therapeutic benefits. Although GLP-1 RAs have primarily been approved for T2D and obesity, they have been shown to reduce cardiovascular risk and mortality [3] and are increasingly recognized for their ability to reduce liver fat and improve hepatic steatosis in animal models as well as in several clinical studies [4,5,6]. GLP-1 RAs are renowned for their effectiveness in promoting weight loss, improving glycemic control, suppressing appetite, and slowing gastric emptying.

Several recent studies have emphasized that the effects of GLP-1 RAs can be sex-specific [7]. Several clinical trials have shown greater weight loss in women with or without diabetes after GLP-1 RA treatment, and the effect of GLP-1 RA treatment on glucose homeostasis may also be sex-dependent, although this is less consistent [8]. In addition, menopause in women, which is typically linked to weight gain, visceral adiposity and an increased risk of developing diabetes, may influence the efficacy of GLP-1 RA treatment. Preliminary clinical research suggests that short semaglutide treatment has a similar effect on fat loss in menopausal women as in premenopausal women [9]. On the other hand, animal research involving ovariectomized females [10] revealed that liraglutide treatment showed promise in restoring lipid homeostasis and preventing weight gain in females with induced menopause. These studies emphasize the significant interaction between GLP-1 and estrogens in regulating lipid and energy metabolism [11].

The metabolic benefits of GLP-1 RAs for women have not been consistently investigated, and it is not entirely clear whether sex could influence the pharmacological action of GLP-1 RAs. Further research is needed to determine their role in preventing metabolic syndrome and prediabetes in menopausal women. In addition, the results of clinical studies may be limited by the inclusion of both pre- and postmenopausal women in the same group. The prevalence and severity of non-alcoholic fatty liver disease (NAFLD) differ significantly between the sexes. Although the overall prevalence is higher in men, postmenopausal women are at a significantly greater risk of severe disease progression [12].

Currently, no experimental studies compare the effects of treatment on sex and reproductive age. Understanding the underlying mechanisms of differences in the effectiveness of GLP-1 RAs according to sex and reproductive age may lead to more accurate and personalized care for people suffering from obesity and diabetes, as well as for those with prediabetes. In this study, we investigated the effect of liraglutide administration on metabolic disorders associated with prediabetes in a prediabetic model without obesity or fasting hyperglycemia, and compared the treatment effect according to the sex and reproductive status of females. The hereditary hypertriglyceridemic rat strain (HHTg), as a non-obese model of prediabetes exhibiting insulin resistance, impaired glucose tolerance, severe dyslipidemia and fatty liver, was used [13,14]. We studied prediabetic males, fertile females and ovariectomized females mimicking menopause. Our previous study [15] demonstrated that ovariectomy-induced menopause in HHTg females exacerbates insulin resistance and impaired glucose tolerance, increasing visceral adiposity and ectopic lipid deposition.

## 2. Materials and Methods

### 2.1. Ethical Approval and Sample Size

In the study, the hereditary hypertriglyceridemic (HHTg) rat strain provided by the Institute for Clinical and Experimental Medicine, Prague, Czech Republic, was used as a non-obese prediabetic model. All experiments were performed in agreement with the Animal Protection Law of the Czech Republic (359/2012) and the Directive 2010/63EU of the European Parliament and of the Council and approved by the Ethics Committee of the Institute for Clinical and Experimental Medicine (protocol number 26/2025, dated 30 May 2025).

The study was performed using 6-month-old male, female and ovariectomized (OVX) female HHTg rats. Six groups were formed: male, female, and OVX—with two groups from each category, which were either treated or untreated. A total of 46 HHTg rats were included in the study. The number of animals in the experimental group was set at *n* = 7 for male groups and *n* = 8 for female groups in order to adhere to the 3Rs (“Replacement, Reduction, Refinement”) principle and to ensure that the necessary sample size for a high-quality statistical analysis was met. The experimental unit was a single animal.

### 2.2. Animals and Diet

Ovariectomized female rats were used as a model mimicking menopause. At 4 months of age, HHTg females were bilaterally ovariectomized under anesthesia with ketamine (70 mg/kg b.wt) and xylazine (10 mg/kg b.wt) administered intraperitoneally. During the procedure, the females were saturated with oxygen followed by subcutaneous analgesia (meloxicam 1 mg/kg b.wt). After surgery, the health status of females was monitored daily. Rats were housed in cages in a room with controlled temperature (22–25 °C), humidity (55–60%), and natural light conditions (12 h/12 h light/dark cycle) and with free access to food (maintenance diet for rats and mice; Altromin, Lage, Germany) and drinking water. The nutrient composition of the maintenance diet includes 19% protein, 11% fat, and 65% carbohydrates. The animals were monitored daily for their overall health, behavior, and postoperative recovery.

HHTg rats were randomly assigned to experimental groups (*n* = 7, resp. 8 in each group, together 46 in total) without or with liraglutide treatment (subcutaneously 0.2 mg/kg/day for 8 weeks). The liraglutide dose was administered subcutaneously every day between 3 and 4 p.m. Randomization was performed by the principal investigator, who was blinded to group assignments, and the animals were randomly assigned to groups. During the liraglutide administration, body weight and food intake were measured weekly. At the end of the experiment, rats aged 8 months were sacrificed after being given light anesthesia (zoletil 5 mg/kg b.wt.) in a non-fasted state. Aliquots of serum and tissue samples were collected, immediately frozen in liquid nitrogen, and stored at −80 °C for further analysis.

### 2.3. Biochemical Analysis

Serum levels of glucose, TAGs, and non-esterified fatty acids (NEFAs) as well as total and HDL cholesterol were measured using commercially available kits (Erba Lachema, Brno, Czech Republic, and FUJIFILM Wako, Neuss, Germany). Serum insulin, glucagon and leptin concentrations were determined using rat ELISA kits (Mercodia AB, Uppsala, Sweden; BioVendor, Brno, Czech Republic; Yanaihara, Awakura, Japan, respectively). Circulating TNFα as well as MCP-1 and adiponectin concentrations were measured using rat ELISA kits (Bio-Source International, San Diego, CA, USA; eBioscience, Beder, Austria, respectively). Serum 17b-estradiol was determined using an ultra-sensitive estradiol RIA kit (Immunotech, Prague, Czech Republic). For the oral glucose tolerance test (oGTT), blood glucose was determined after a glucose load (300 mg/100 g b.wt.) administered intragastrically after six hours of fasting. Blood was drawn from the tail before the glucose load at 0 min (baseline) and 30, 60, 120 and 180 min thereafter.

To determine TAGs and cholesterol in tissues (liver—*large hepatic lobe*, myocardium—*left ventricle*, and skeletal muscle—*gastrognemius*), samples were extracted in chloroform/methanol (2:1, *v*/*v*). A solution of 2% potassium dihydrogenphosphate was then added to the mixture and centrifuged; the organic phase formed from the mixture was evaporated under N_2_. The resulting pellet was dissolved in isopropyl alcohol, with TAG or cholesterol content determined by an enzymatic assay (Erba-Lachema, Brno, Czech Republic). To determine diacylglycerols (DAGs) in the liver, samples were extracted in dichloromethane/methanol (1:1, *v*/*v*). The dissolution of the resulting pellet in isopropyl alcohol was followed by isolation by thin-layer chromatography. The amount of separated DAGs was determined by an enzymatic assay (Roche Diagnostics, Mannheim, Germany).

As a marker of skeletal muscle insulin sensitivity, basal and insulin-stimulated glycogen synthesis was determined ex vivo in the isolated *musculus soleus* by measuring the incorporation of ^14^C-U glucose into glycogen as previously described [16]. The basal and insulin-stimulated lipid synthesis in visceral adipose tissue was determined by measuring the incorporation of ^14^C-U glucose into lipids in isolated epididymal and perimetrial adipose tissue. The release of NEFA into the incubation medium with adrenaline (0.25 μg/mL) was used to measure adrenaline-stimulated lipolysis in the epididymal or perirenal adipose tissue ex vivo. The concentrations of NEFA in the medium were determined using an available kit (FUJIFILM Wako, Lexington, MA, USA).

### 2.4. Fatty Acid Composition and Fatty Acid Desaturase Activity

For determination of fatty acid (FA) composition in phospholipids in the liver, samples were extracted in dichloromethane/methanol (50:50, *v*/*v*). The organic phase was evaporated under N_2_ and the resulting pellet dissolved in an isopropyl alcohol/hexane mixture. Phospholipid classes were separated by thin-layer chromatography using hexane–diethyl ether–acetic acid (70:30:1, *v*/*v*) as a solvent system, then extracted from silica gel, and finally converted to fatty acid methyl esters (FAMEs). FAMEs were established by gas chromatography (Hewlett-Packard GC system) with hydrogen as the carrying gas, a flame ionization detector, and a carbowax-fused silica capillary column (Varian, Polo Alto, CA, USA) [17]. Peaks for individual FAs were identified by comparing their retention times with those of authentic standards (mix of fatty acids, Restek, Bellefonte, PA, USA). The percentage of each FA relative to the total fatty acids analyzed was then reported. The activity of fatty acid desaturases was determined by calculating the product-to-precursor ratio to account for the enzymatic activity involved in fatty acid metabolism: delta-5 desaturase (20:4n6/20:3n6) and delta-6 desaturase (18:3n6/18:2n6). The anti-inflammatory index was calculated as the selected PUFA ratio (22:6n3 + 22:5n3 + 20:3n6 + 20:5n3)/20:4n6 [18].

### 2.5. Oxidative Stress Markers

A HPLC method with fluorescence detection was used to determine the levels of reduced (GSH) and oxidized (GSSG) glutathione forms in the liver. This method was performed using a HPLC diagnostic kit (ChromSystems, Gräfelfing, Germany). The activity of the antioxidant enzymes superoxide dismutase and glutathione peroxidase was analyzed using Cayman Chemicals assay kits (Ann Arbor, MI, USA) according to spectrophotometric measurement of enzyme substrates or products. The activity of SOD was analyzed using the reaction blocking nitrotetrazolium blue reduction and nitroformazan formation. The activity of GPx was monitored by oxidation of glutathione using Ellman’s reagent. MDA, a parameter of lipid peroxidation, was assessed by the HPLC method with fluorescence detection (HPLC diagnostic kit, ChromSystems, Germany).

### 2.6. Relative mRNA Expression

Total RNA was isolated from liver tissue using RNA Blue (Top-Bio, Prague, Czech Republic). RNA quality and concentration were determined spectrophotometrically (Nanodrop One, Thermo Fisher Scientific, Waltham, MA, USA). Reverse transcription was performed with an EvoScript Universal cDNA Master Kit (Roche, Basel, Switzerland). The quantitative real-time PCR was performed on 1536-well-format plates using the acoustic liquid handler Echo 550 (Labcyte, Dublin, Ireland) and LightCycler 1536 Instrument (Roche, Basel, Switzerland). Relative expressions were determined after normalization against *Hprt1* as the internal reference and calculated using the 2^−ΔΔCt^ method. The reference gene Hprt1 was selected for normalization based on its demonstrated stability across various tissues. Results were run in quadruplicate.

### 2.7. Statistical Analysis

Two-way ANOVA was used to analyze the individual and combined effects of treatment (P treatment) and treatment interactions according to sex and reproductive state in the HHTg strain (P interaction). To determine sample size, power analysis was done using R version 4.5.0. *A priori* power analysis (α = 0.05, power = 0.80) confirmed that *n* = 7 per experimental group was sufficient to detect treatment effects. All analyzed data were normally distributed according to the Shapiro–Wilk test. Tukey’s post hoc test was used for variables showing evidence of treatment interactions depending on sex (P sex, between males and females) or the reproductive status of HHTg females (P menopause, between females and ovariectomized females). The test was adjusted for multiple comparisons to determine whether liraglutide treatment would significantly influence metabolic parameters in HHTg males, females or OVX females. One-way ANOVA with Fisher’s LSD post hoc test was used to determine liraglutide’s effect on hepatic gene expression in HHTg males. Statistical significance was determined at a value of *p* < 0.05. All results are expressed as mean ± SEM. Statistical analysis was performed using GraphPad Prism10.

### 2.8. Description of Data Management

Data obtained from the analysis of samples will be collected on an ongoing basis and stored in a shared folder accessible only to members of the project’s research team. The data will be stored on the research institution’s servers.

## 3. Results

**Figure 1:**
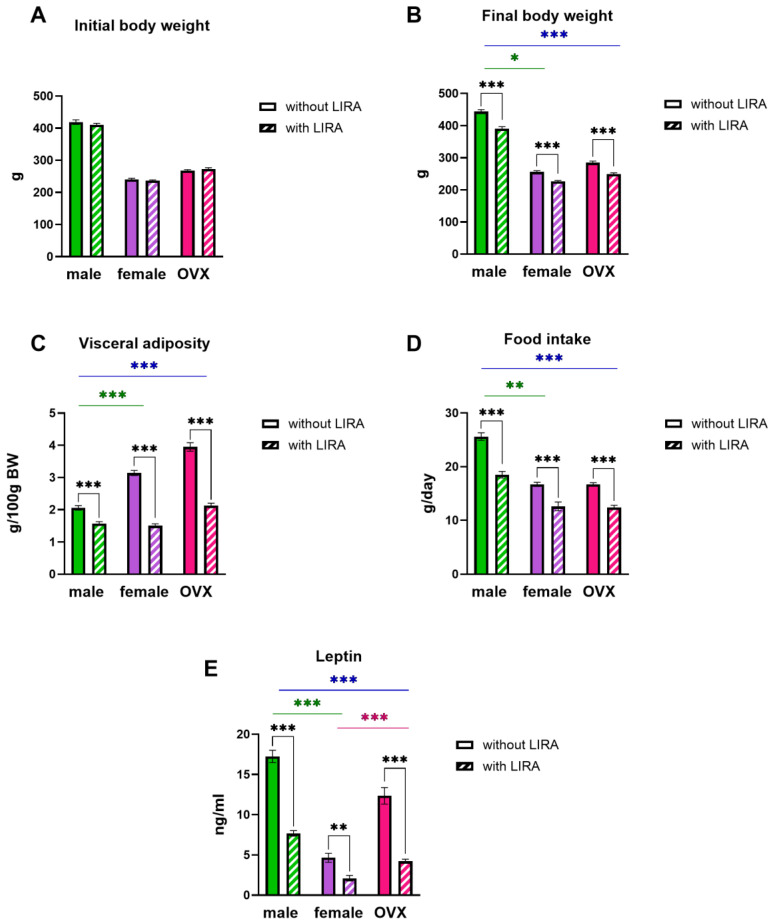
Effects of liraglutide on initial body weight (**A**), final body weight (**B**), adiposity (**C**), food intake (**D**) and serum leptin levels (**E**) in male, female and OVX female HHTg rats. Data are expressed as mean ± SEM, *n* = 7 for male groups and *n* = 8 for female groups, analyzed by two-way ANOVA with Tukey’s post hoc test; P_treatment_—significance reflecting liraglutide treatment across all groups (blue); P_sex_—significance reflecting treatment interactions depending on sex between male and female (green) HHTg rats; P_menopause_—significance reflecting treatment interactions depending on reproductive status of females, between female and OVX female (red) HHTg rats. * denotes *p* ˂ 0.05, ** denotes *p* < 0.01, *** denotes *p* < 0.001; OVX—ovariectomized female rats.

Before liraglutide administration, there were no significant differences in initial body weight, glucose or serum TAGs between the untreated and treated groups of HHTg rats—male, female and OVX. Liraglutide administration markedly decreased food intake (25% reduction, *p* ˂ 0.001) (Figure 1) but had no effect on drink intake.

### 3.1. Effects of Liraglutide on Body Weight and Basal Characteristics According to Sex and Reproductive Status

Liraglutide administration resulted in significant reductions in body weight and adiposity index in all experimental groups (Figure 1). In addition, ovariectomized females had a higher body weight and visceral adiposity than females of reproductive age. Liraglutide administration did not affect the relative weight of brown adipose tissue (BAT) in fertile or ovariectomized females. Treatment also significantly decreased serum leptin levels in all groups, which corresponds to decreased food intake, regardless of sex or reproductive status. The decrease in leptin levels after liraglutide administration was greater in ovariectomized females (*p* ˂ 0.001) than in females of reproductive age (*p* ˂ 0.01) (Figure 1). As shown in Table 1, liraglutide treatment had a favorable influence on serum lipids: circulating TAG levels decreased markedly while HDL-cholesterol levels increased. The effect of liraglutide on serum TAG levels differed significantly between HHTg males and females, and this effect persisted even after menopause. The effect on HDL cholesterol was observed only in HHTg females of reproductive age and females with induced menopause. In addition, liraglutide administration decreased NEFA in both HHTg males and females (Table 1). In contrast, serum total cholesterol concentrations were unaffected in all experimental groups. Decreased pro-inflammatory serum levels of TNFα were observed in liraglutide-treated HHTg female rats, while the serum concentrations of adiponectin and MCP-1 remained unchanged in all experimental groups (Table 1). There were no significant changes in serum estradiol concentrations after liraglutide administration.

### 3.2. Effects of Liraglutide on Glucose Metabolism and Insulin Sensitivity in Peripheral Tissue and in the Liver According to Sex and Reproductive Status

**Figure 2:**
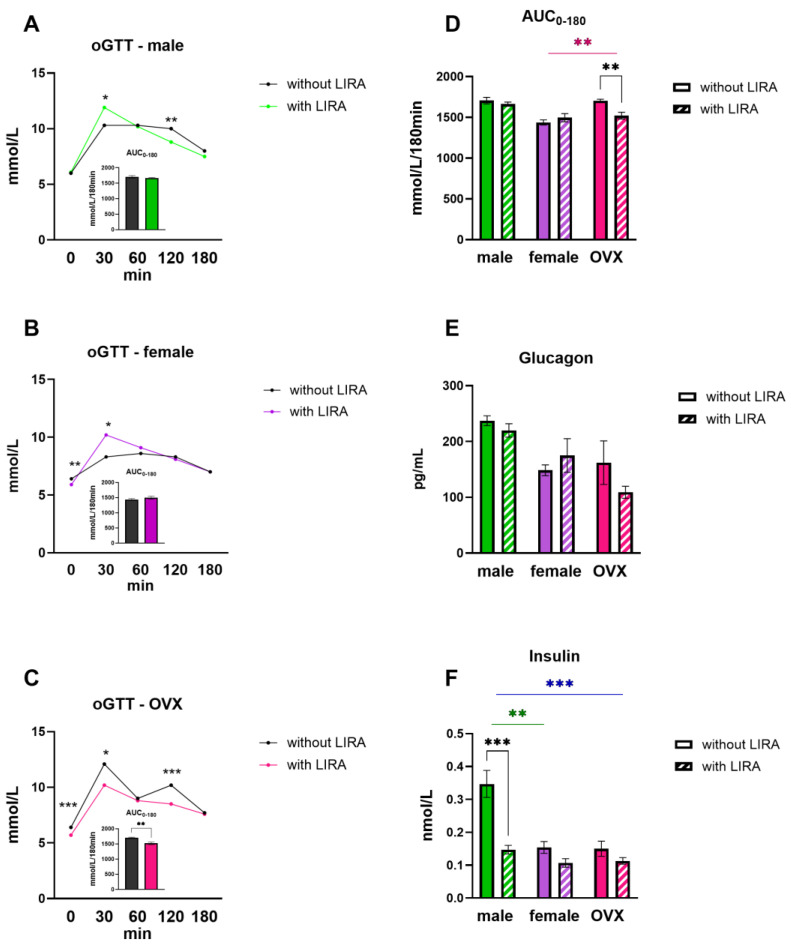
Effects of liraglutide on the oGTT test in male (**A**), female (**B**), and OVX female (**C**) HHTg rats, and on AUC (**D**), glucagon (**E**) and insulin (**F**) in male, female and OVX female HHTg rats. Data are expressed as mean ± SEM, *n* = 7 for male groups and *n* = 8 for female groups, analyzed by two-way ANOVA with Tukey’s post hoc test; P_treatment_—significance reflecting liraglutide treatment across all groups (blue); P_sex_—significance reflecting treatment interactions depending on sex between male and female (green) HHTg rats; P_menopause_—significance reflecting treatment interactions depending on reproductive status of females, between female and OVX female (red) HHTg rats. * denotes *p* ˂ 0.05, ** denotes *p* < 0.01, *** denotes *p* < 0.001; OVX—ovariectomized female rats; AUC—area under the curve during the oral glucose tolerance test.

**Figure 3:**
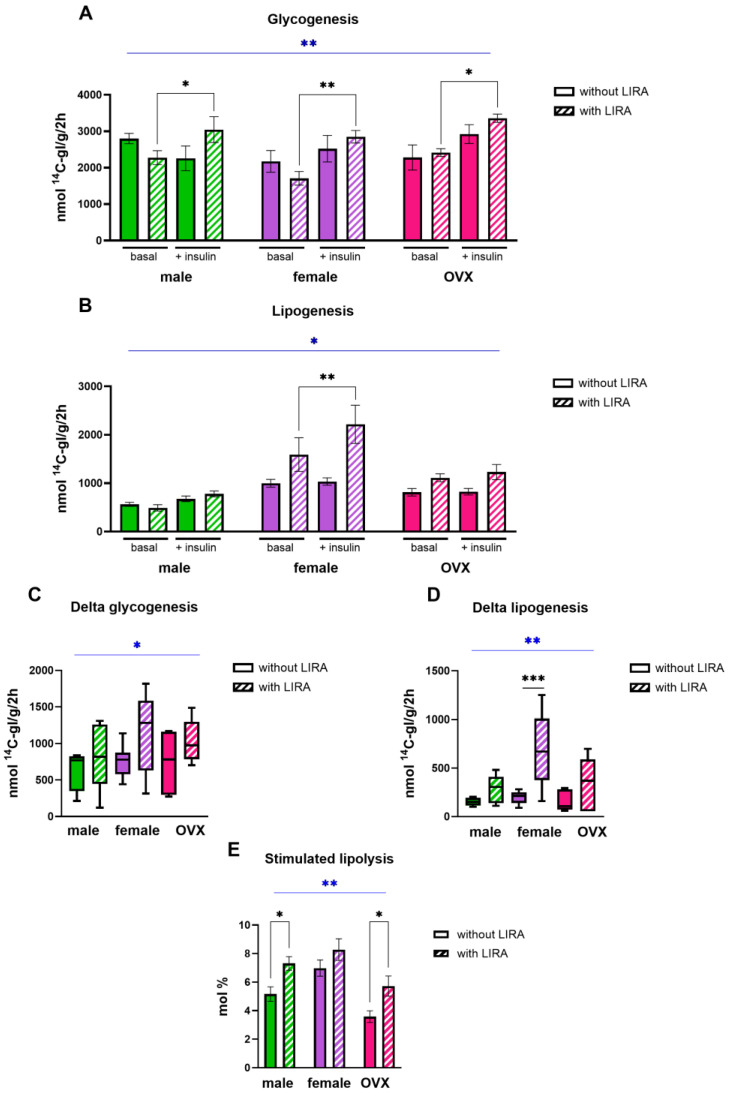
Effects of liraglutide on the markers of insulin sensitivity in the skeletal muscle (**A**)—basal and insulin-stimulated glycogenesis and in visceral adipose tissue (**B**)—basal and insulin-stimulated lipogenesis, (**C**)—delta glycogenesis, (**D**)—delta lipogenesis and adrenaline-stimulated lipolysis (**E**) in male, female and OVX female HHTg rats. Data are expressed as mean ± SEM, *n* = 7 for male groups and *n* = 8 for female groups, analyzed by two-way ANOVA with Tukey’s post hoc test; P_treatment_—significance reflecting liraglutide treatment across all groups (blue); P_sex_—significance reflecting treatment interactions depending on sex between male and female HHTg rats (green); P_menopause_—significance reflecting treatment interactions depending on reproductive status of females, between female and OVX female (red) HHTg rats. * denotes *p* ˂ 0.05, ** denotes *p* < 0.01, *** denotes *p* < 0.001; OVX—ovariectomized female rats.

As shown in Figure 2, liraglutide treatment improved glucose tolerance and altered the oGTT curve in all groups, as assessed by oGTT. In addition, liraglutide significantly reduced the AUC_0-180_ in ovariectomized females (*p* ˂ 0.01) (Figure 2). Serum insulin levels were reduced in the group of males, while serum glucagon levels remained unchanged in all groups of the HHTg strain. Liraglutide treatment was associated with increased insulin sensitivity parameters in both skeletal muscle and visceral adipose tissue, measured as basal and insulin-stimulated glucose incorporation into muscle glycogen (glycogenesis) or lipids of visceral adipose tissue (lipogenesis) (Figure 3). Liraglutide increased insulin-stimulated glycogenesis in all groups of the HHTg strain. Delta glycogenesis or delta lipogenesis, which highlight the differences between basal and insulin-stimulated glycogenesis or lipogenesis, increased following liraglutide administration. Furthermore, treatment with liraglutide led to a significant improvement in insulin sensitivity, particularly in skeletal muscle and visceral adipose tissue in females of reproductive age. Adrenaline-stimulated lipolysis from visceral adipose tissue was significantly elevated in all experimental groups after liraglutide administration (Figure 3).

**Figure 4:**
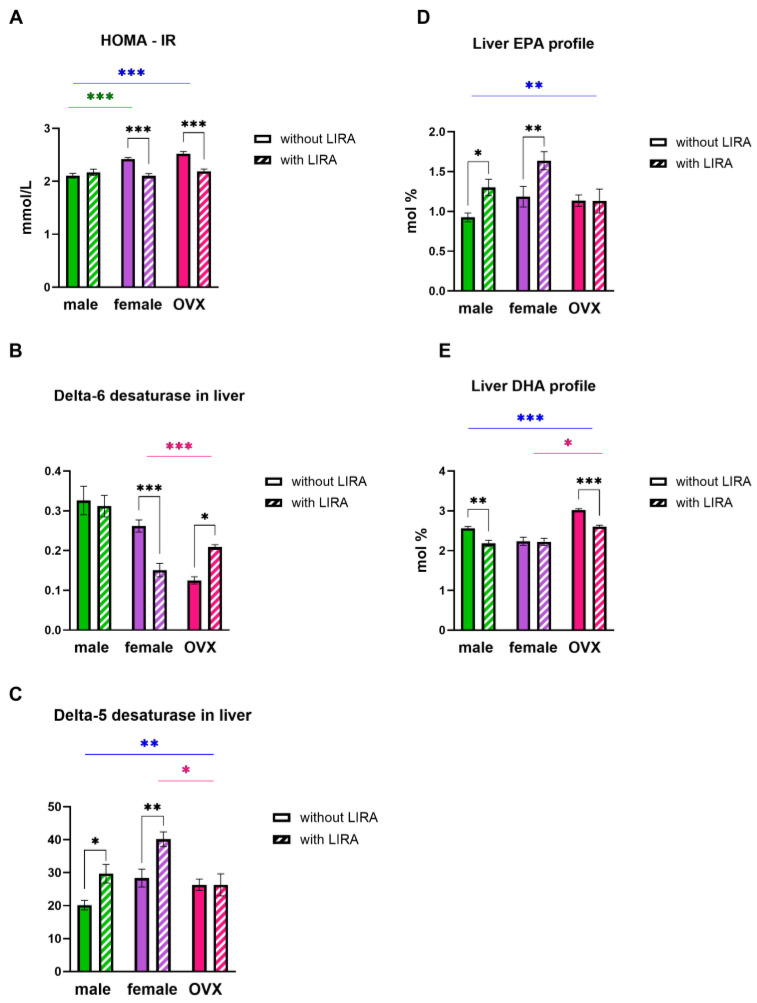
Effects of liraglutide on HOMA-IR (**A**), delta-6 desaturase (**B**), delta-5 desaturase (**C**), and the profiles of EPA (**D**) and DHA (**E**) in the liver in male, female and OVX female HHTg rats. Data are expressed as mean ± SEM, *n* = 7 for male groups and *n* = 8 for female groups, analyzed by two-way ANOVA with Tukey’s post hoc test; P_treatment_—significance reflecting liraglutide treatment across all groups (blue); P_sex_—significance reflecting treatment interactions depending on sex between male and female (green) HHTg rats; P_menopause_—significance reflecting treatment interactions depending on reproductive status of females, between female and OVX female (red) HHTg rats. * denotes *p* ˂ 0.05, ** denotes *p* < 0.01, *** denotes *p* < 0.001; HOMA-IR—Homeostatic Model Assessment for Insulin Resistance; EPA—eicosapentaenoic acid; DHA—docosahexaenoic acid.

Liraglutide administration decreased HOMA-IR (Homeostatic Model Assessment for Insulin Resistance) in HHTg females of reproductive age as well as in ovariectomized female rats (*p* ˂ 0.001), whereas no reduction was observed in HHTg males (Figure 4). Reduced hepatic delta-6 desaturase together with elevated hepatic delta-5 desaturase can improve insulin sensitivity in the liver, with the most significant effects observed in HHTg females of reproductive age. According to the FA profile in hepatic phospholipids, liraglutide administration changed the n-3 PUFA profile in the liver (Figure 4). The profile of EPA increased while the profile of DHA decreased after liraglutide treatment.

### 3.3. Effects of Liraglutide on Ectopic Lipid Accumulation and Hepatic Gene Expression Profile Involved in Lipid Metabolism According to Sex and Reproductive Status

**Figure 5:**
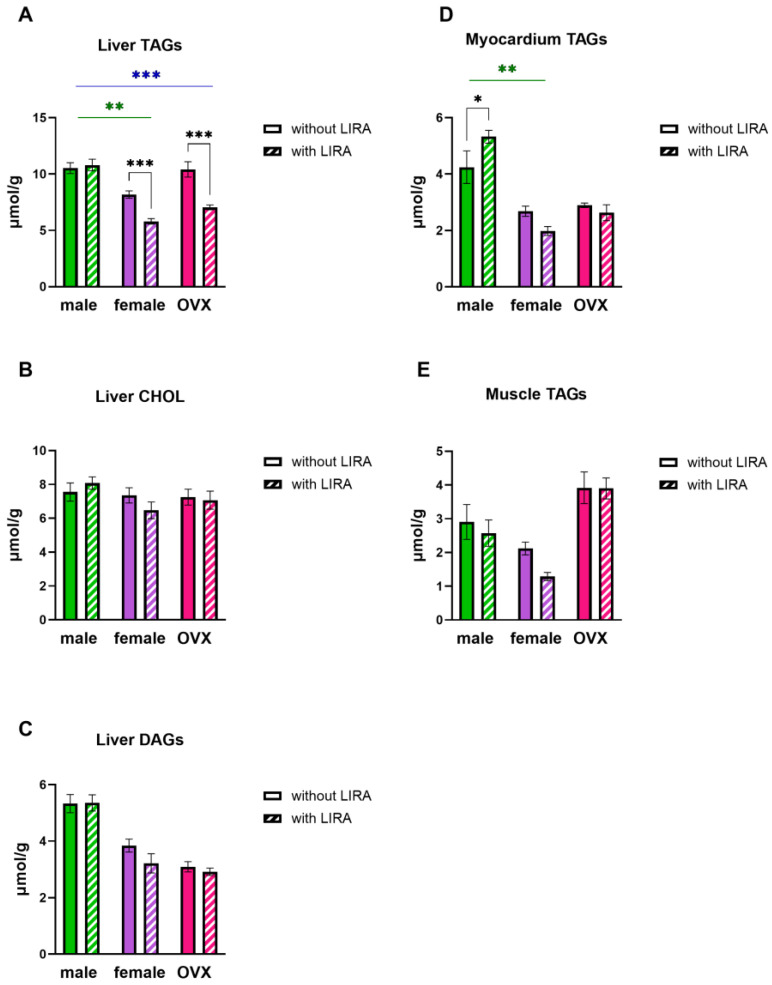
Effects of liraglutide on ectopic TAGs (**A**), cholesterol (**B**) and DAGs (**C**) accumulation in the liver and ectopic TAGs accumulation in myocardium (**D**) and skeletal muscle (**E**) in male, female and OVX female HHTg rats. Data are expressed as mean ± SEM, *n* = 7 for male groups and *n* = 8 for female groups, analyzed by two-way ANOVA with Tukey’s post hoc test; P_treatment_—significance reflecting liraglutide treatment across all groups (blue); P_sex_—significance reflecting treatment interactions depending on sex between HHTg male and female (green) HHTg rats; P_menopause_—significance reflecting treatment interactions depending on reproductive status of females, between female and OVX female (red) HHTg rats. * denotes *p* ˂ 0.05, ** denotes *p* < 0.01, *** denotes *p* < 0.001; OVX—ovariectomized female rats; TAGs—triacylglycerols; CHOL—cholesterol; DAGs—diacylglycerols.

As shown in Figure 5, liraglutide administration significantly reduced ectopic TAG accumulation in the liver in female HHTg rats (*p* ˂ 0.001), and this effect persisted after ovariectomy. However, hepatic TAG accumulation in male rats was unaffected. There were no significant differences in hepatic cholesterol or lipotoxic diacylglycerols between male, female and OVX female HHTg rats after liraglutide treatment (Figure 5). TAG accumulation in the myocardium increased in male rats after liraglutide treatment, whereas it remained unchanged in female rats of reproductive age and in ovariectomized females. Liraglutide did not affect TAG accumulation in skeletal muscle in any of the experimental groups of HHTg rats. There was a trend towards decreased TAG accumulation in the skeletal muscles of HHTg females of reproductive age, although the reduction did not reach statistical significance (Figure 5).

**Figure 6:**
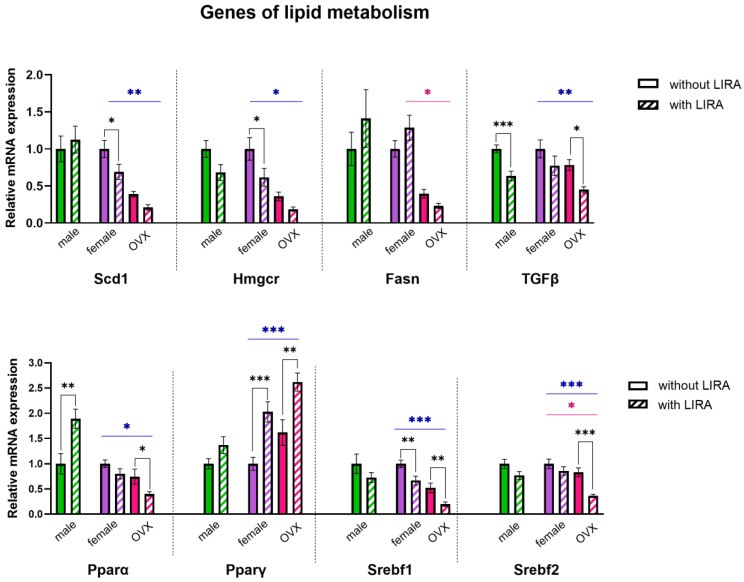
Effects of liraglutide on mRNA gene expression of enzymes and transcription factors in the liver in male, female and OVX female HHTg rats. Data are expressed as mean ± SEM, *n* = 7 for male groups and *n* = 8 for female groups, analyzed by one-way ANOVA with Fisher’s LSD post hoc test; P_treatment_—significance reflecting liraglutide treatment across all groups (blue); P_sex_—significance reflecting treatment interactions depending on sex between male and female (green) HHT rats; P_menopause_—significance reflecting treatment interactions depending on reproductive status of females, between female and OVX female (red) HHTg rats. * denotes *p* ˂ 0.05, ** denotes *p* < 0.01, *** denotes *p* < 0.001. OVX—ovariectomized female rats; SCD1—stearoyl-coenzyme A desaturase 1; HMGCR—3-hydroxy-3-methylglutaryl-coenzyme A reductase; FASN—fatty acid synthase; TGFβ—transforming growth factor beta; PPARα—peroxisome proliferator-activated receptor alpha; PPARγ—peroxisome proliferator-activated receptor gamma; SREBF1,2—sterol regulatory element-binding protein 1,2.

As shown in Figure 6, liraglutide significantly altered the relative gene expression of enzymes and transcription factors involved in energy and lipid metabolism, with the most pronounced effect observed in HHTg females of reproductive age. Decreased hepatic mRNA expression of *Scd1*, *Hmgcr* and *Srebf2* following liraglutide administration reduced lipogenesis and cholesterol synthesis, which may contribute to reduced hepatic lipid accumulation. Markedly increased mRNA expression of *Pparγ* and reduced mRNA expression of *Srebf1* after liraglutide administration can participate in both lipid metabolism and insulin sensitivity in the liver. These effects of liraglutide were clearly observed in HHTg females and partially in HHTg OVX females, but not in HHTg males (Figure 6). On the other hand, liraglutide significantly reduced hepatic mRNA expression of *Tgfβ*, which can contribute to improvement of hepatic fibrosis and steatosis.

### 3.4. Effects of Liraglutide on Hepatic Oxidative Stress and Inflammation According to Sex and Reproductive Status

**Figure 7:**
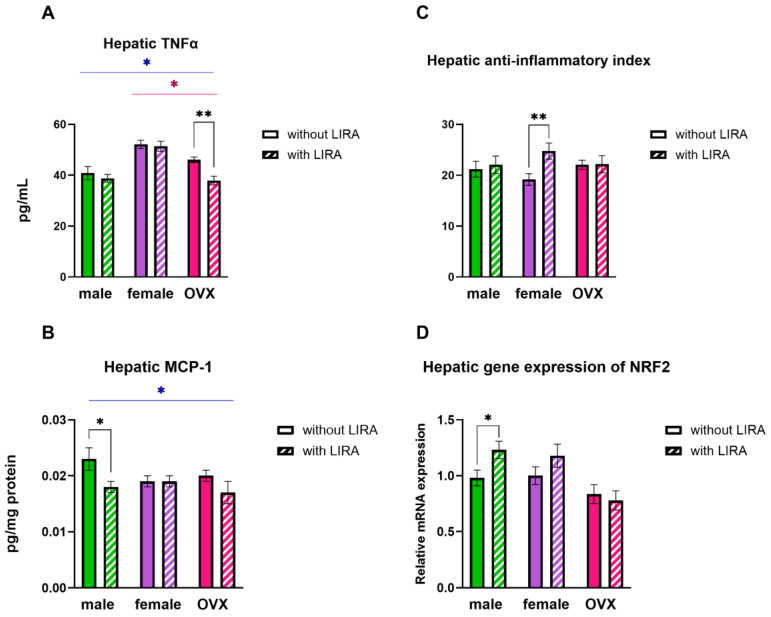
Effects of liraglutide on hepatic inflammatory parameters TNFα (**A**), MCP-1 (**B**), anti-inflammatory index (**C**) and mRNA gene expression of Nrf2 (**D**) in HHTg male, female and OVX female rats. Data are expressed as mean ± SEM, *n* = 7 for male groups and *n* = 8 for female groups, analyzed by two-way ANOVA with Tukey’s post hoc test; P_treatment_—significance reflecting liraglutide treatment across all groups (blue); P_sex_—significance reflecting treatment interactions depending on sex between male and female (green) HHTg rats; P_menopause_—significance reflecting treatment interactions depending on reproductive status of females, between female and OVX female (red) HHTg rats. * denotes *p* ˂ 0.05, ** denotes *p* < 0.01. OVX—ovariectomized female rats; TNFα—tumor necrosis factor alpha; MCP-1—monocyte chemoattractant protein-1; Nrf2—nuclear factor erythroid-2-related factor 2; the anti-inflammatory index was calculated as the selected PUFA ratio (22:6n3 + 22:5n3 + 20:3n6 + 20:5n3)/20:4n6.

Liraglutide administration significantly improved the parameters of oxidative stress in the liver (Table 2). The ratio of the reduced to oxidized form of glutathione (GSH/GSSG) increased in the liver, with a more pronounced effect observed in females and ovariectomized females. Additionally, liraglutide significantly decreased the oxidized form of glutathione in all experimental groups. The elevated activity of the glutathione-dependent antioxidant enzyme GPx following liraglutide treatment may contribute to the increased GSH/GSSG ratio. However, liraglutide increased the gene expression of the Nrf2 transcription factor in the liver only in the male group. Furthermore, decreased TNFα and MCP-1 concentrations in the liver after liraglutide treatment can reduce chronic hepatic inflammation (Figure 7). An increase in the anti-inflammatory index, which is expressed as the ratio of selective pro- and anti-inflammatory polyunsaturated fatty acids, may reduce inflammation in the liver.

## 4. Discussion

The results of this study support the idea that the GLP-1 agonist liraglutide, independent of its glucose-lowering effects, may have positive effects on certain metabolic disorders associated with prediabetes, such as hepatic steatosis, insulin resistance, and lipid metabolism. The effectiveness of these effects may be influenced by sex, and in females, it can also change after menopause. Furthermore, the absence of body weight obesity in the prediabetic rat model makes the presented results independent of their direct anti-obesity effects.

As expected, liraglutide administration has a favorable effect on glucose tolerance and insulin sensitivity of peripheral tissues. While glucose homeostasis improved regardless of sex or the presence of menopausal status, liraglutide administration most significantly enhanced insulin sensitivity in fertile females, particularly in visceral adipose tissue. This may be due to the influence of sex hormones, as well as to a more significant reduction in visceral adiposity. Another mechanism that may contribute to improved insulin sensitivity in females is the greater reduction in NEFA levels. On the other hand, a reduction in insulinemia in prediabetic males was associated with an equally substantial decrease in leptin levels, suggesting a potential link with leptin resistance. In line with our findings, the insulin-sensitizing effect of GLP-1 RA was observed in mice with diabetes induced by a high-fat diet [19]. The beneficial effects of GLP-1 RAs on pancreatic β-cells and increased insulin secretion have been demonstrated [20,21], but the indirect reduction in body weight may contribute to the improvement in insulin sensitivity. Clinical studies suggest that GLP-1 RAs may improve insulin sensitivity more pronouncedly in women, but the results are not yet consistent [8]. This may be due to the fact that in clinical studies the group of women is not divided into pre- and postmenopausal subgroups. Our results confirm that a significant increase in insulin sensitivity occurred, particularly in fertile females.

In line with clinical studies [22], our study observed a significant 10% reduction in body weight after liraglutide treatment, despite the use of a non-obese prediabetic model. The body weight-lowering effect of liraglutide was associated with a significant reduction in visceral adiposity in all groups. The most significant reduction in visceral adiposity was observed in the group of fertile females. Interestingly, this reduction persisted in females even after ovariectomy, and this reduction was more pronounced than in males. Weight loss and reduction of visceral adiposity correspond to a 25% decrease in food intake and a marked decrease in leptin levels. GLP-1 RAs influence and regulate food intake via a central dual effect, acting on both anorexigenic signaling and the dopaminergic neurotransmitter system [23]. According to our results, this effect is very pronounced and consistent across all groups. In addition to regulating food intake, liraglutide was found to significantly increase adrenaline-stimulated lipolysis in visceral adipose tissue. This may contribute to a reduction in visceral adiposity, which is followed by weight loss. The results of this study indicate that metabolism in adipose tissue after liraglutide administration is more affected in females than in males. Estrogen receptors may contribute to this, as they provide a neuroanatomical basis for the possible interaction between GLP1-RA activation, sex, and estrogens. In women, estradiol is essential not only for reproductive functions, but also for regulating food intake and energy metabolism. The anorexigenic effect of GLP-1 RA after central injection has been attributed to GLP-1 RA-expressing targets in the hypothalamus and brainstem [24], as has the anorexigenic effect of estrogens, which act in the same regions of the brain’s nervous system [25].

In our previous study [15], we found that ovariectomy led to significant changes in lipid metabolism in adipose tissue, and that estrogen substitution could partially reverse some of these metabolic complications. The effect of GLP-1 RAs on weight loss is significant and expected, but their influence according to sex or menopausal status is unclear. The study by Model et al. [11] emphasizes the direct effect of GLP-1 RAs on adipose tissue in female rats and ovariectomized rats and highlights the complexities of the interactions between GLP-1 and estrogen in regulating metabolism. Our results are consistent with human studies indicating that liraglutide is effective at reducing weight in patients with obesity, but interestingly, better results are reported in those with insulin resistance and prediabetes [26]. Greater weight loss was also observed in patients with MASLD [27].

Human studies suggest that sex can influence the response to anti-obesity therapy [28]. Despite limited evidence indicating greater weight loss in women undergoing anti-obesity pharmacotherapy, sex-specific analysis remains underexplored. In our study, liraglutide’s effects on body composition and insulin sensitivity differed between prediabetic male and female HHTg rats. The direct influence of sex hormones and different pharmacodynamics may both contribute. Pharmacokinetic analysis of liraglutide showed 32% higher exposure in women than in men, independent of body weight [28,29]. The promotion of weight loss is an effect of the female sex as an independent factor. Sex hormones may influence the action of GLP-1 RAs and their efficacy [8]. In addition, the effects may vary depending on the menopausal status of females, with their effectiveness potentially decreasing after menopause. Reports suggest that estrogen increases total GLP-1 secretion from human pancreatic and intestinal cells, suggesting that sex may influence endogenous responses to GLP-1 [8,11].

In our study, liraglutide’s effect on dyslipidemia was examined in a non-obese prediabetic rat strain that exhibited severe hypertriglyceridemia and chronically elevated NEFA [30,31]. Recent studies have shown that certain GLP-1 receptor agonists, such as liraglutide and semaglutide, have a beneficial effect on serum lipid levels. It has been found that treatment with GLP-1 agonists is associated with a reduction in serum TAGs and cholesterol in diabetic and obese patients, as well as in obese mice with NAFLD [32,33,34]. Although no significant differences in the effect on NEFA levels were observed with liraglutide [35], our results indicated a reduction in serum NEFA levels in both male and female groups. Females experienced a greater reduction, which led to greater insulin sensitivity. A positive correlation has been observed between levels of TAGs and total cholesterol and the degree of abnormal lipid metabolism. A meta-analysis [36] shows that in many studies, treatment with liraglutide leads to a reduction in TAG and total cholesterol levels, which is similar to our results. In HHTg rats, liraglutide reduced serum TAG levels in all groups; however, total cholesterol levels were not affected. The elevated serum HDL-cholesterol levels observed in both female groups following liraglutide treatment suggest an increased cholesterol efflux capacity, which may offer some protection against hypercholesterolemia, hepatic cholesterol accumulation, and lipotoxicity [37].

No accumulation of cholesterol in the liver was detected in any group. However, the reduction in the *Hmgcr* pathway, together with a reduction in the transcription factor related to cholesterol metabolism, *Srebp2*, suggests a beneficial effect of liraglutide on reducing liver fat and managing dyslipidemia, with a more pronounced effect in both female groups.

This study also demonstrated the significant effect of liraglutide on hepatic lipid deposition. The accumulation of hepatic TAGs decreased in female rats, regardless of their reproductive age. Meanwhile, TAG accumulation in male rats remained unchanged. The mechanism underlying the hypolipidemic effect of liraglutide on the liver is most likely due to decreased lipogenesis and reduced lipid transport into cells. However, the hepatic gene expression of the enzyme involved in fatty acid synthase *Fasn* was unaffected in all groups. Nevertheless, the decrease in the relative gene expression of *Scd1* in the liver supports the idea of lower de novo lipogenesis. Reduction in *Scd1* can result in increased fatty acid oxidation, decreased fat storage, and improved insulin sensitivity in the liver. In addition, liraglutide administration appears to enhance insulin sensitivity by reducing liver lipid deposition via downregulation of *Srebp1*, a key regulator of hepatic lipogenesis. Improvements in lipid metabolism in the liver were observed in female groups, with the greatest effect seen in females of fertile age. In the male group, liraglutide did not alter lipid accumulation or the relative gene expression involved in lipid metabolism. This may be related to estrogen levels, which significantly affect hepatic lipid metabolism by promoting lipid oxidation and inhibiting TAG synthesis and fatty acid uptake [38].

MASLD is a major cause of liver disease worldwide [1]. Several animal models and clinical studies have demonstrated that GLP-1RAs can reduce hepatic steatosis, inflammation and fibrosis [39,40]. Postmenopausal women have a higher risk of developing MASLD and its progression. In general, premenopausal women have a lower risk of cardiometabolic, cognitive and hepatic disease. However, they lose this protection after menopause, resulting in a disease prevalence that is comparable to or higher than that of men [41]. In this study, administration of liraglutide increased insulin sensitivity in the liver and decreased endogenous glucose production, resulting in an improvement in hepatic steatosis. Improvement in liver function is probably the result of a combination of total weight loss and direct metabolic effects in the liver, such as the suppression of de novo lipogenesis and the restoration of hepatic insulin sensitivity. The effect is either direct or indirect, through either weight reduction or improved insulin resistance. This may contribute to the suppression of inflammation and fibrosis, thereby reversing the development of MASLD. In line with some studies [42,43], liraglutide was also found to reduce the hepatic expression of *TGF-β*, which plays an important role in improving liver fibrosis.

A reduction in HOMA-IR, together with reductions in delta-6 desaturase and increases in delta-5 desaturase in the liver, may improve hepatic insulin sensitivity and alleviate hepatic steatosis. Furthermore, the effect of the transcription factors *Pparγ* and *Srebf1*, together with an increase in the n-3 PUFA EPA profile, may contribute to this improvement. In the liver *Pparγ* reduces chronic inflammation and plays a role in regulating glucose homeostasis, thereby affecting hepatic insulin sensitivity [44]. *Pparα* is also an important factor in regulating insulin sensitivity and lipid metabolism. Upregulation of *Pparα* reduces weight gain and lipid accumulation through its activation of AMPK signaling increasing fatty acid oxidation. In this study, liraglutide increased *Pparγ* expression in females, contributing to greater improvement in insulin sensitivity, while it increased *Pparα* expression in males. Liraglutide affects these parameters across experimental groups. Its impact on those associated with MASLD is most evident in the fertile female group. However, ovariectomized females also exhibit a significant effect on HOMA-IR and reduced liver TAG accumulation.

Although the protective effects of GLP-1 RAs on hepatic steatosis are closely linked to their effect on weight loss, the underlying mechanism is likely pleiotropic, suggesting that they may reduce hepatic lipid accumulation independently of weight loss [45]. GLP-1 RAs may directly affect hepatic lipid storage by improving hepatic glucose metabolism, promoting lipolysis and increasing fatty acid oxidation, as well as improving hepatic insulin resistance [46]. In addition to reducing lipid accumulation in the liver, other possible mechanisms include alleviating the oxidative or endoplasmic reticulum stress response and reducing hepatic chronic inflammation. This study observed a slight reduction in chronic liver inflammation and oxidative stress in the liver, particularly in both female groups. Glutathione, which correlates well with the accumulation of TAGs in the liver, was found to improve hepatic oxidative stress after GLP-1 RA treatment. The anti-inflammatory effects of liraglutide may be related to its reduction of pro-inflammatory cytokines. However, the positive effect of liraglutide on the pro- and anti-inflammatory fatty acid composition observed in our study may also contribute to these effects. In accordance with our findings, GLP-1 RAs have been shown to significantly reduce serum levels of IL-6 and TNFα in diabetic patients [46]. In a study involving diabetic mice [47], liraglutide was found to significantly improve hepatic steatosis by downregulating the TNFα inflammatory signaling pathway directly in the liver; however, only male mice were used in this study.

A recent meta-analysis has demonstrated the efficacy and safety of liraglutide in reducing the severity of NAFLD in patients with or without comorbid T2D, but the analysis did not consider sex differences [6]. Our prediabetic rats showed that females have better liver lipid reduction than males, even after menopause. However, after menopause, HHTg female rats do not respond as strongly to treatment as female rats of fertile age. This amplifying effect in females is partially attributed to synergistic interactions with estrogens, which could influence the pharmacological action of GLP-1 RAs. Therefore, estrogen can enhance the antidiabetic actions of GLP-1 RAs. Both hormones, GLP-1 and estrogen, can regulate the release of each other and can have similar effects. The results of some clinical studies examining the differences in the effects of liraglutide on men and women are limited by the fact that the women in the study were of an average age typical of menopause, with some in the premenopausal period and some in the postmenopausal period.

This study has some limitations that should be acknowledged. Firstly, the main limitation of this study is that the estrous cycles of fertile females were not synchronized prior to sacrifice. Fluctuations in sex hormones can influence some metabolic parameters, including lipids, and are also responsible for the higher deviation (SEM) of measured parameters in the group of fertile females. Secondly, liraglutide significantly reduced food intake in all experimental groups, meaning that some of its effects may be secondary and related more to weight loss than to a direct pharmacological effect. However, it is difficult to separate these effects, and they are likely to complement each other. Finally, in the study the interpretation of gene expression is based on mRNA expressions with the absence of expressions of protein levels.

## 5. Conclusions

The results suggest that sex and reproductive age could be vital factors in determining the efficacy of liraglutide with regard to its metabolic effects. In a prediabetic model, liraglutide treatment improved glucose tolerance independently of sex and reproductive age. However, prediabetic females experienced a reduction in hepatic lipid accumulation and exhibited greater improvements in insulin sensitivity and hepatic lipid metabolism compared to prediabetic males. Both fertile and ovariectomized females observed a favorable effect of liraglutide on hepatic lipid accumulation and visceral adiposity; however, the beneficial effect on insulin sensitivity in prediabetic females was reduced after ovariectomy. Our findings suggest that the response to liraglutide treatment may vary according to sex and reproductive age, emphasizing the need for an individualized approach in people with prediabetes.

## Figures and Tables

**Table 1:** Effects of liraglutide on basal metabolic characteristics.

	Male	Male LIRA	Female	Female LIRA	OVX	OVX LIRA	P_Treatment_	P_Interaction_
Fasting glucose (mmol/L)	6.0 ± 0.1	6.1 ± 0.1	6.4 ± 0.0	5.9 ± 0.2 **	6.4 ± 0.0	5.7 ± 0.1 ***	<0.001	<0.05
Serum TAGs (mmol/L)	4.03 ± 0.40	2.57 ± 0.46 *	7.50 ± 0.58	2.35 ± 0.26 ***	3.86 ± 0.41	1.37 ± 0.18 ***	<0.001	<0.001
Serum cholesterol (mmol/L)	1.87 ± 0.04	1.67 ± 0.07	1.88 ± 0.16	1.68 ± 0.06	1.91 ± 0.06	2.15 ± 0.05	n.s.	<0.05
HDL cholesterol (mmol/L)	0.62 ± 0.01	0.62 ± 0.01	0.44 ± 0.01	0.71 ± 0.02 ***	0.66 ± 0.02	0.83 ± 0.03 ***	<0.001	<0.001
NEFA (mmol/L)	0.73 ± 0.07	0.50 ± 0.05 **	0.63 ± 0.05	0.32 ± 0.06 ***	0.62 ± 0.05	0.48 ± 0.05	<0.001	n.s.
Adiponectin (μg/mL)	0.39 ± 0.03	0.35 ± 0.05	0.38 ± 0.04	0.30 ± 0.04	0.39 ± 0.03	0.38 ± 0.05	n.s.	n.s.
Estradiol (pg/mL)	n.d.	n.d.	41.91 ± 3.97	48.94 ± 6.50	22.46 ± 1.25	22.27 ± 1.54	n.s.	n.s.
TNFα (pg/mL)	11.49 ± 0.92	11.85 ± 0.76	12.62 ± 0.68	7.79 ± 0.64 *	10.17 ± 0.96	8.15 ± 0.64	<0.05	n.s.
MCP-1 (ng/mL)	7.38 ± 1.41	5.93 ± 0.87	8.85 ± 1.73	9.18 ± 1.96	6.17 ± 1.10	5.35 ± 0.67	n.s.	n.s.

Two-way ANOVA results: P_treatment_ denotes the significance of liraglutide treatment; P_interaction_ denotes the significance of liraglutide in all groups (treatment vs. group interaction). For multiple comparisons Tukey’s post hoc test was used; * denotes *p* ˂ 0.05; ** denotes *p* ˂ 0.01; *** denotes *p* ˂ 0.001. Data are expressed as mean ± SEM; *n* = 7 for male groups and *n* = 8 for female groups. OVX—ovariectomized females; TAGs—triacylglycerols; NEFA—non-esterified fatty acid; TNFα—tumor necrosis factor α; MCP-1—monocyte chemoattractant protein; n.s.—not significant, n.d.—not determined.

**Table 2:** Effects of liraglutide on oxidative stress parameters in the liver.

	Male	Male LIRA	Female	Female LIRA	OVX	OVX LIRA	P_Treatment_	P_Interaction_
GSH (mmol/mg protein)	62.0 ± 2.4	61.7 ± 2.3	66.4 ± 1.8	69.6 ± 1.4	69.4 ± 1.2	66.6 ± 1.8	n.s.	n.s.
GSSG (mmol/mg protein)	1.05 ± 0.08	0.81 ± 0.05 *	0.84 ± 0.06	0.56 ± 0.04 **	1.18 ± 0.12	0.70 ± 0.08 ***	<0.001	n.s.
GSH/GSSG	61.4 ± 4.2	77.1 ± 4.3	80.9 ± 4.0	130.2 ± 4.5 ***	62.6 ± 6.6	101.4 ± 9.8 ***	<0.001	<0.05
MDA (nmol/mg protein)	2.24 ± 0.17	2.54 ± 0.10	2.45 ± 0.11	2.38 ± 0.13	2.21 ± 0.14	2.05 ± 0.12	n.s.	n.s.
SOD (U/mg protein)	66.4 ± 4.8	72.3 ± 4.1	46.6 ± 5.1	43.7 ± 3.7	41.1 ± 4.7	39.1 ± 3.3	n.s.	n.s.
GPx (mmol NADPH/min/mg protein)	88.5 ± 4.3	131.7 ± 8.3 *	93.2 ± 9.2	167.7 ± 20.2 **	70.3 ± 8. 9	101.2 ± 14.3	<0.001	n.s.

Two-way ANOVA results: P_treatment_ denotes the significance of liraglutide treatment; P_interaction_ denotes the significance of liraglutide in all groups (treatment vs. group interaction). For multiple comparisons Tukey’s post hoc test was used; * denotes *p* ˂ 0.05; ** denotes *p* ˂ 0.01; *** denotes *p* ˂ 0.001. Data are expressed as mean ± SEM; *n* = 7 for male groups and *n* = 8 for female groups. OVX—ovariectomized females; GSH—reduced form of glutathione; GSSG—oxidized form of glutathione; MDA—malondialdehyde; SOD—superoxide dismutase; GPx—glutathione peroxidase; n.s.—not significant.

## Data Availability

All data generated during this study are included in the article.

## Abbreviations

The following abbreviations are used in this manuscript:

DAG	diacylglycerol
DHA	docosahexaenoic acid
EPA	eicosapentaenoic acid
FASN	fatty acid synthase
GLP1-RA	glucagon-like peptide-1 receptor agonist
GSH	reduced form of glutathione
GSSG	oxidized form of glutathione
HMGCR	3-hydroxy-3-methylglutaryl-coenzyme A reductase
HOMA-IR	Homeostatic Model Assessment for Insulin Resistance
4-HNE	4-hydroxynonenale
IL-6	interleukin 6
MDA	malondialdehyde
MASLD	metabolic dysfunction-associated steatotic liver disease
MCP-1	monocyte chemoattractant protein-1
NAFLD	non-alcoholic fatty liver disease
NEFA	non-esterified fatty acid
NRF2	nuclear factor erythroid-2-related factor 2
OVX	ovariectomized females
PPARα	peroxisome proliferator-activated receptor alpha
PPARγ	peroxisome proliferator-activated receptor gamma
SCD1	stearoyl-coenzyme A desaturase 1
SREBP1	sterol regulatory element-binding protein 1
TAG	triacylglycerol
TGFβ	transforming growth factor beta
TNFα	tumor necrosis factor alpha
